# On the cognitive bases of illusionism

**DOI:** 10.7717/peerj.9712

**Published:** 2020-08-25

**Authors:** Jordi Camí, Alex Gomez-Marin, Luis M. Martínez

**Affiliations:** 1 Universitat Pompeu Fabra, Barcelona, Spain; 2 Instituto de Neurociencias CSIC-UMH, Alicante, Spain

**Keywords:** Magic, Cognition, Real-world neuroscience

## Abstract

Cognitive scientists have paid very little attention to magic as a distinctly human activity capable of creating situations that are considered impossible because they violate expectations and conclude with the apparent transgression of well-established cognitive and natural laws. This illusory experience of the “impossible” entails a very particular cognitive dissonance that is followed by a subjective and complex “magical experience”. Here, from a perspective inspired by visual neuroscience and ecological cognition, we propose a set of seven fundamental cognitive phenomena (from attention and perception to memory and decision-making) plus a previous pre-sensory stage that magicians interfere with during the presentation of their effects. By doing so, and using as an example the deconstruction of a classic trick, we show how magic offers novel and powerful insights to study human cognition. Furthermore, live magic performances afford to do so in tasks that are more ecological and context-dependent than those usually exploited in artificial laboratory settings. We thus believe that some of the mysteries of how the brain works may be trapped in the split realities present in every magic effect.

## Introduction

Illusionism is a millenary art whose social context has evolved substantially over time. Currently it is a pure form of entertainment, but there was a time when it was related to the activities of the priests, the mediums, the sorcerers and witches (Scot, 1584). Contemporary magic is unique in the sense that it is capable of provoking the wonderful experience of the impossible without the need for the audience to actually believe that what they observe is real (we use “magic” and “illusionism” as interchangeable concepts). With that in mind, illusionists have mastered a myriad of methods and techniques that have withstood the test of time. These techniques have been perfected using purely empirical means, just by trial-and-error, and have been preserved and, up until now, transmitted with discretion amongst the pundits. The wisdom of magicians is such that it encompasses many scientific disciplines, including physics (especially optics, but also mechanics and electronics), new materials, mathematics, and, above all and mainly, cognitive science.

It is remarkable how little attention has been traditionally paid to magic (Tompkins, 2020), not only scientifically but also as a distinctly human activity capable of creating events that are considered impossible because they violate expectations, concluding with the apparent transgression of natural laws. It is as remarkable, if not more, that this is so despite the fact that magic techniques appeal to a set of seven fundamental cognitive phenomena, from attention and perception to memory and decision-making and a previous pre-sensory stage. For the purposes of this review, we understand by cognitive phenomena not only low-level mechanisms and processes but also those high-level tasks or operations that the brain executes continuously to (1) process the information we receive from the environment, (2) put it in context of our previous experiences and other previously acquired knowledge, so that (3) we can analyze reality and interact with it adequately. Cognitive phenomena that also enable us to (4) be flexible and adapt our behavior to the changes and demands of different situations. Our personal experience as magicians and an extensive review of the cognitive and magic literature allowed us to establish the cognitive bases that underlie magicians’ ability to induce the illusion of the “impossible” at the climax, or outcome, of many magic effects. Impossible outcomes generate in the audience an initial surprise that reflects the cognitive dissonance between what is expected and what is actually perceived. This dissonance is followed by a more complex, subjective and highly diverse set of reactions that are known as the “magical experience”. Some may experience pleasure in response to the dissonance, while others may react with discomfort or even anger (Teller, 2015; Leddington, 2016). We provide a detailed description of the seven main cognitive phenomena, and a very relevant previous pre-sensory stage, that magicians can interfere with during the presentation of any magic effect. We concentrate exclusively on the cognitive phenomena behind the impossible experience induced by illusionist magicians, we do not enter into the realm of the magical experience. In this context, we sustain the putative controversial claim that any magic effect will involve manipulations that never reach the sensory stage and at least some of the identified seven cognitive phenomena, and only those.

We do not attempt to define *cognitive phenomena* simply in terms of their neural correlates, since in that regard we still know next to nothing about the illusion of impossibility; recently, Caffaratti et al. (2016), have measured for the first-time specific changes in brain activity through electrophysiological records during the magician’s manipulations. In keeping with this, the few neuroimaging experiments that have so far measured changes in brain dynamics during the observation of magic effects have found that the same brain areas previously reported during problem solving and conflict monitoring tasks light up when the illusion of impossibility is experienced (Parris et al., 2009; Danek et al., 2015). But magic is, perhaps rather uniquely, a social, relational process. Magicians do not react to the observation of their own effects (Danek et al., 2015). In other words, magicians are probably the only artists who cannot trick themselves. Musicians can enjoy their own performance; for magic to exist, for the illusion of impossibility to occur, however, it is imperative that there be at least one spectator.

**Figure 1:**
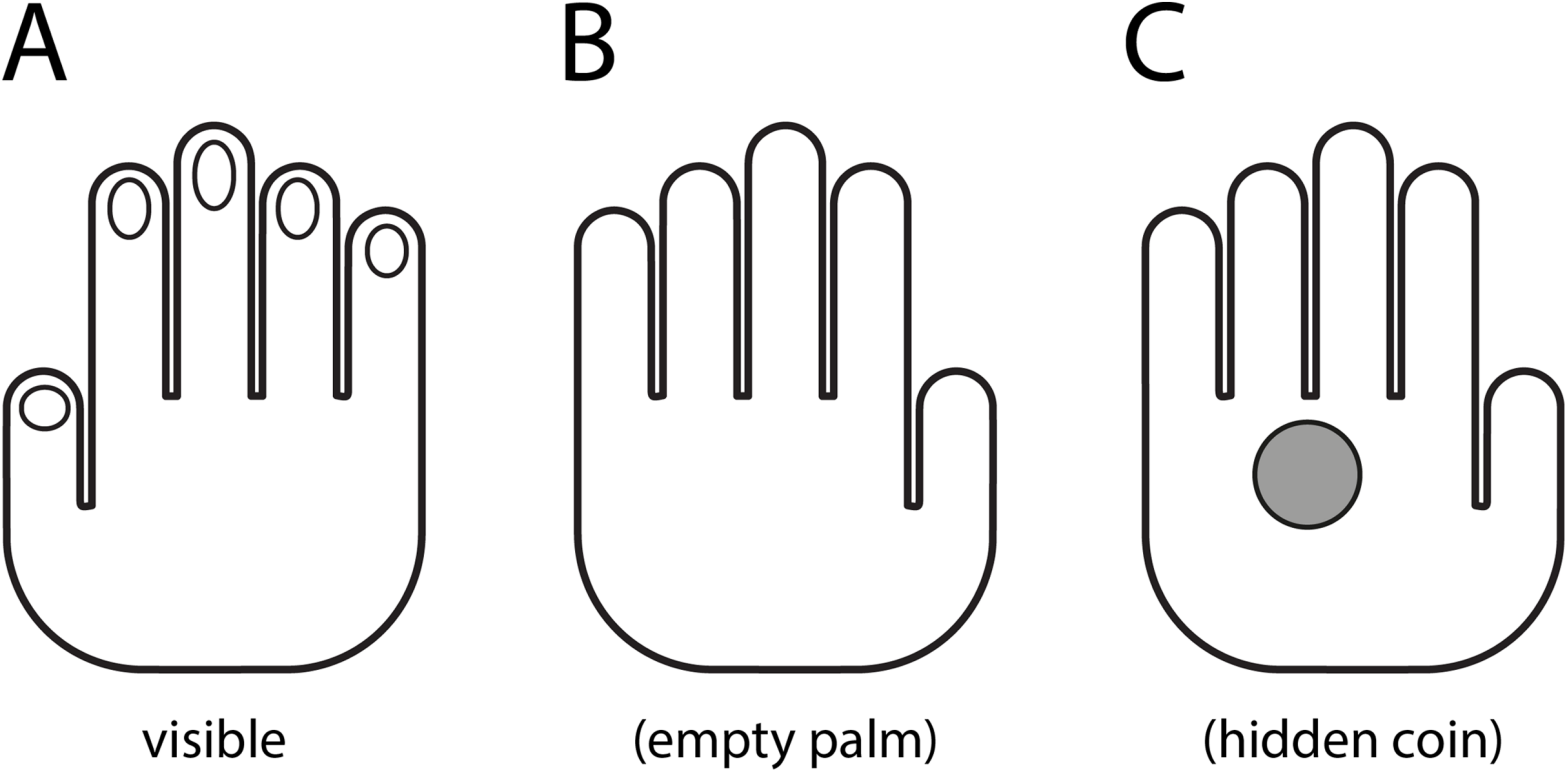
Magic exploits different affordances and splitted Umwelts. (A) Sketch of the (visible) dorsal side of a magician’s hand. (B) The (invisible) palm side of the magician’s hand from the subjective perspective of a naive spectator. (C) The (invisible) palm for a spectator that knows the trick.

Our proposal draws both from ecological psychology (Gibson, 1979) and enactivism (Clark, 2008; Varela, Thompson & Rosch, 1991) as scientific approaches that seek to go beyond the orthodoxy of cognitivism. The magicians’ gestures and maneuvers, the gimmicks and objects they use and all the backdrop of a magical effect, including its plot, work because they *afford* meaningful perceptions to the audience. Coined by Gibson, an *affordance* is defined as the quality of an object (say, a coin or a hand, but also more abstract objects, like a story) that defines its possible uses or makes clear how it can or should be used. An affordance is thus always relational, namely, it entangles each perceiving subject with their perceived object in the world. As a corollary, affordances of the same object can vary dramatically across subjects. For instance, a tree for an ant has very little to do with a tree for a carpenter (Gomez-Marin, 2019a). During a magic trick, whether the spectator actually makes use of the properties of the objects that the magician presents, or simply imagines their possible uses, they nevertheless constitute opportunities for action. The magician’s job is to control the perception in order to induce certain affordances that direct the audience to the desired impossible outcome. Under this perspective, the cognitive foundation for magic must also be found in ecologically available information in the environment, and not only in the way it relates to individual internal perceptions. For most of us, an open hand with the back facing forward (Fig. 1A) affords an empty hand (Fig. 1B). However, for the magician (or for a spectator that knows the trick), it affords an opportunity to inadvertently hide and transfer a coin or card (Fig. 1C) constituting the secret behind the effect. In line with key biosemiotic principles (Von Uexküll, 1926), the same aspect of the “physical surroundings” (the *Umgebung*), the open hand, can thus provide a different “meaningful environment” (the *Umwelt*) to a lay spectator and to another magician in the audience, and even to the same individual at different points in time. In the magician’s hand, at least two different *Umwelts* (*Umwelten* in proper German) coexist, that of the fellow magicians who know the maneuvers, and that of the lay spectators who do not. The oblivion that all organisms share the world but not all organisms have the same world in common produces both in cognitive neuroscientists and in magic spectators a critical blind spot (Gomez-Marin, 2019b).

Related to this, magic does not work the same in laboratory conditions and during real live sessions. Affordances are context-dependent, the environment is a constitutive, not a mere enabling part of cognition (Gibson, 1979; Gomez-Marin & Ghazanfar, 2019). In fact, the few experiments that, to date, compared the same effects by the same magician in both situations yielded quite different results (Shalom et al., 2013). The same is true when other aspects of human cognition, such as the perception of beauty and artistic expression are judged directly in a museum and not in the aseptic laboratory environment (Leder & Nadal, 2014). The problem is that it is all too common for experiments outside the laboratory to sacrifice precision, accuracy and control for ecological relevance, making the comparisons difficult. However, magic, as we shall see, offers a very promising and virtually unexplored solution. With their ancient tradition, magicians have developed very controlled, perfectly repeatable routines that work for practically one hundred percent of their audience, without exception. Their effects interfere, either in isolation or concurrently, with almost all the cognitive processes studied in the laboratory, from attention and memory to perception and decision-making, and they do it without sacrificing control for ecological relevance. Since the nineteenth century, most researchers have approached magic trying to understand its foundations from the postulates and techniques of psychology and cognitive science. The time is ripe to make the path in the opposite direction and take advantage of the enormous resources that magic offers to understand cognitive phenomena using less reductionist and more integrative approaches and with great ecological and behavioral significance.

While new magic effects, compatible with current technological developments, are constantly being introduced, many classic effects have survived our own cultural and technical evolution and continue to be made as they were centuries ago with equal success, simply because they rely on pre-sensory manipulations or touch upon very relevant and universal cognitive phenomena. Cups and balls, for instance, a magic trick already described by Seneca two thousand years ago is very different from the 19th century Robert-Houdin’s effect in which he simultaneously lit thousands of candles in the theater. While the former works and survives because it depends exclusively on our perceptual and motor priors (evolutionary ingrained), the latter depends on the audience’s ignorance of the existence of electricity (culturally assimilated). The former continues to amaze us today, the second is no longer included in magic shows.

Thus, we propose that the magic techniques that generate the illusion of impossibility are successful only because they take advantage of the strategies that we use to overcome our important limitations of capacity, extended processing time and relatively large energy requirements. As we will show, these techniques interfere with the basic mechanisms of sensory processing, attention, perception, memories as well as those of intuitive, learned and/or innate, decisions and behaviors. They exploit our strong unconscious biases and expose the automatisms and predispositions that characterize the functioning of our brains. They create, manipulate, transform or hide our perceived affordances and influence even our simple interactions with the physical and social world; and they do it very efficiently, unlike most of our cognitive experiments in the lab, they work in every show and virtually for every spectator in the audience. However, the potential of harnessing these powerful magic techniques in research, especially in designing experiments with great ecological relevance, has been very scarcely exploited, probably due to a great mutual ignorance, and even suspicion, between the two fields.

## Survey Methodology

Our literature review includes more than a hundred references, the majority of which are specifically devoted to magic. As mentioned in the introduction, we have concentrated on the illusion of impossibility as performed by illusionist magicians (mainly close-up magic tricks). As mentioned above, we will not deal with how the audience feels and reasons after the climax of the effect, nor with other magic disciplines. We have not included popular books on the matter, nor scientific articles on magic that did not touch on cognition (such as mathematics or computational science applied to magic). Other criteria for exclusion of articles were as follows. In using search engines, one comes across the following challenge: the term “magic”, which is an extraordinary polysemic word, retrieves close to three million results in Google. This includes businesses, novels and films, music and sports, theories of consciousness, pickpocketing, and the supernatural. None of these is the focus of our manuscript. Other search terms used were “magician”, “illusion”, and “illusionism”. In order to ensure a comprehensive and unbiased coverage of such literature that was focused at the same time, we used PubMed and Google Scholar, as well as references contained in classic papers, books, and reviews on the matter. Our search was also guided by two extra sources. On the one hand, we have thoroughly consulted journals and books on magic outside the strict academic discipline, such as the online catalog (AskAlexander.org) from the Conjuring Arts Research Center (NY), which is deemed the most complete library on conjuring arts. Moreover, we have been in direct conversations with a wide range of Spanish professional magicians. Finally, it may come as a surprise that there are not even one hundred references in total when it comes to the scientific study of the science of magic (Tompkins, 2020). One may compare this with the thousands of papers that are published every year in nearly any discipline and sub-discipline of cognitive science. We do not claim to have cited them all here (nor was it our purpose), but we are convinced that our survey represents virtually everything that there is to know on the pre-sensory and cognitive phenomena that lead to the illusion of impossibility in magic up to the present day.

### The structure of a magic effect

A magic effect may tap into practically all the cognitive processes studied in the laboratory. In general, every magic effect has the following structure: a presentation or demonstration of an expository nature—with or without a plot or storyline—that ends with a climax or magic upshot. The duration of a magic effect can range from seconds to a few minutes. A magic game can contain one or more effects, and a magic routine consists of a set of effects or consecutive games. The magic effects that are the subject of this article are those that are performed live and produce the illusion of impossibility in the audience, in contrast to those seen on television or laboratory computers and whose impact is generally much lower.

A central but seldom recognized conception in magic is that in every effect two different worlds coexist in parallel. According to the Spanish magician Arturo de Ascanio, the first world is called the “external life” of the effect and it consists of what the audience consciously experiences, sees, hears and touches. It is the “emptiness” of the open hand in Fig. 1B. The second world, or the “internal life”, includes everything that the magician secretly manipulates as the effect progresses (Etcheverry, 2000). It is the fact that there is a hidden coin in the palm of the hand in Fig. 1C. To achieve the illusion of impossibility it is necessary for the magician to coherently combine the obvious and patent actions of the “external life”, the affordances they provide, with the concealments, secret maneuvers and the use of various gimmicks and gadgets, that hide other affordances that live only in the “internal life”. This concept of double or split reality is essential to understand how magicians interact with their audience. And it forms also the basis that justify our proposal about the need of studying magic in ecological conditions (Gibson, 1979).

Without spectators there is no magic. Through their actions, magicians generate in the audience an *Umwelt* (Von Uexküll, 1926) providing a unique set of *affordances* (Gibson, 1979) that, during the trick, only magicians control, leading the spectators to anticipate a solution which will, in the end, be frustrated by the great impossible ending. Ascanio used to say that magicians need that all actions of the “external life” have, by themselves, a high degree of likelihood; they want them to follow logical and predictable sequences, without raising any suspicion, until the effect arrives at the magic finale, thus creating the maximum contrast between the initial situation and the outcome of the game.

The most important aspect of the “external life” of the effect is that the structure and the elements of the presentation are all clear, obvious and coherent so that the public can follow their affordances smoothly and be surprised when their expectations are violated at the end. When magicians need to execute an action that does not provide a clear affordance to the audience, or the affordance provided is undesirable, they need to create a new one that establishes a clear and positive relation; and they usually do it by familiarization through repetitive exposure. It is what Ascanio called *conditioned naturalness*.

The essence of the “internal life”, on the other hand, is the skillful concealment of all secret manipulations and related affordances. In Fig. 1C, what the magician conceals is the affordance of the open hand to hold the coin, not the coin. And that is, why magic is known as “the art of hiding the art (*ars artem celandi*)”; which has its maximum expression when, in the climax of an effect, the public expresses in amazement “no way, if the magician has done nothing!”.

### The pre-sensory and cognitive phenomena involved in magic effects

**Figure 2:**
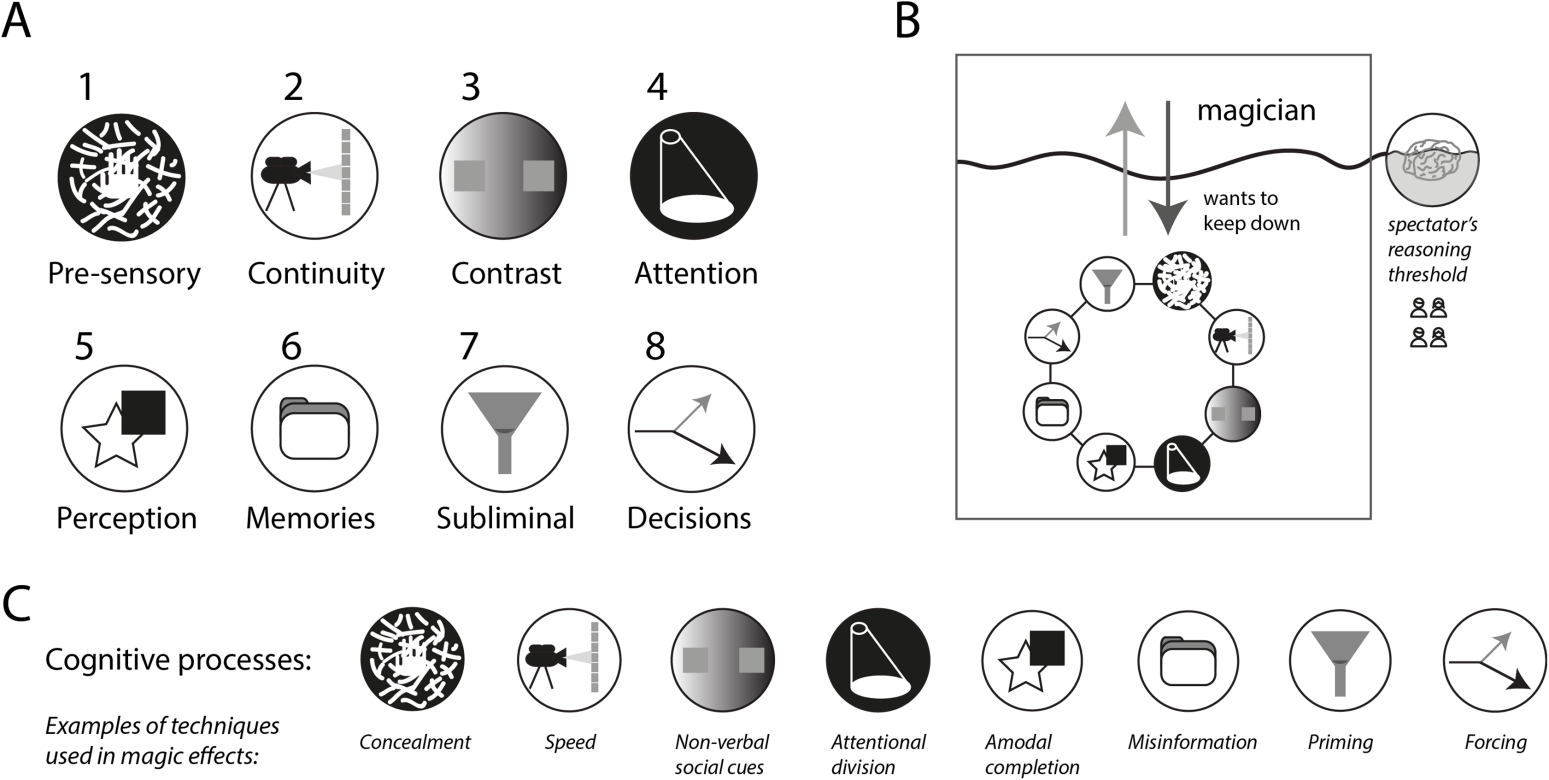
Cognitive phenomena involved in magic effects. (A) We sustain that every magic trick hijacks one or several of these, but only these, cognitive phenomena. (B) In the dynamics between the spectator and the magician, the magician tries to keep all these cognitive phenomena under the spectator’s reasoning threshold. (C) Examples of techniques used in magic effects that correspond to the pre-sensory and cognitive phenomena in (A).

To explain how magic achieves the “illusion of impossibility”, we dissect in different sections the main pre-sensory and cognitive phenomena involved and provide some representative examples of magic effects whose effectiveness rely on them. Most magic techniques and procedures in use today can be assigned to at least one of the eight sections we present below (Fig. 2).

#### Pre-sensory manipulations

A great deal of materials and methods that are used in magic effects involve the concealment or interference with the objects in a scene at a pre-sensory stage, that is, prior to the activation of brain neurons and sense organs. Magic uses concealment very often and does so through very different techniques, such as direct physical covers, optical manipulations, disguise or camouflages. Simple examples of camouflage would be Black Theatre or the use of decks of cards with the black back on a black mat in close-up magic. These pre-sensory manipulations are often combined with visual or cognitive illusions (Macknik et al., 2008) such as when a magician appears to bend a spoon effortlessly simply by rubbing it between the tips of his fingers. Sometimes magician’s resort to the use of auditory masks, such as a certain musical score or a timely sharp noise to cover the sound of a secret contraption at any given time. In the large settings that characterize some “Grand illusions” (stage-magic with large objects), optical tricks are widely used, created through the presentation of black spaces, mirrors and other gadgets, allowing magicians to conceal or distort volumes at a certain distance. In addition to these optical manipulations, “Grand illusions” also benefits from the use of pre-sensory manipulations and visual and cognitive illusions, for instance when the perception of depth is concurrently altered using illusory perspectives.

#### Illusion of continuity

The challenges we face when interpreting even the simplest of scenes are daunting: from the inherently ambiguous information we collect filtered through the veil of our sensory systems, to the relative slowness of our nerve circuits and their characteristically noisy nature, all these make it very difficult to integrate the rapid and continuous flow of information we receive into a coherent, seamless string of percepts and actions. By way of convention, we group under the concept of “illusion of continuity” those cognitive mechanisms that allow us to have a continuous and complete experience, both in space and time. In the case of vision, these include the phenomena of change blindness, our difficulty in perceiving detail at fast presentation speeds, and the processes that contribute to image fusion linked to iconic or sensory memories (Sperling, 1960).

Some magic effects take advantage of the fusion or filling in mechanisms supported by iconic memory. This is the principle underlying Dani DaOrtiz’s rendition of a famous trick by the nineteenth-century Peruvian magician “L’Homme Masqué” (DaOrtiz, 2014). In that effect, the magician riffles in front of a spectator a deck of cards that contains the whole suit of hearts, with the particularity that the Queen of Hearts has been replaced by the Queen of Diamonds. Due to an effect of retinal persistence, this subtle change in symbol, red diamond instead of red heart, at the exposed upper corner of the card is imperceptible for the spectator as the entire red suit is riffled through at a relatively high speed. Magic also takes advantage of the existence of sensory persistence in other modalities, such as touch; that is, the mechanism that allows a pickpocket to remove the watch from a spectator without her noticing. The artist, before and while undoing the strap, exercises light pressure against the wrist of the spectator, thus generating a brief “post-sensation” of still wearing the watch that prevents the astonished victim to realize that, in fact, this has already been stolen (Macknik et al., 2008).

Magic effects also take advantage of those situations in which change blindness occurs (Rensink, O’Regan & Clark, 1997). Change blindness is usually defined as our inability to perceive even very obvious changes in a scene when they are introduced very slowly or after a brief visual interruption. Thus, even large changes in the visual scene can go unnoticed if they coincide with a transitory interruption, such as a blink, an eye movement, flashes on the scene or sudden changes in the direction of movement even when people are looking at the right place (Yao, Wood & Simons, 2019). It has been even observed that fixating and attending to a feature of an object during an effect, for instance a coin, does not guarantee that a change in its identity will be noticed by the spectators (Smith, Lamont & Henderson, 2012). One of the best examples of change blindness in magic is the classic Henry Hardin’s “Princess Card Trick”, an effect that is, found on the web in countless versions (we suggest that of Lance Burton in https://youtu.be/8CvwvskFhTY). In this effect, short-term memory and attention limits prevent the audience to consciously compare and realize that the first and second set of cards are all different.

Another popular example in card manipulation is the Elmsley Count, a technique in which apparently four different cards are shown face up, but in reality, one of them is shown twice, something that always goes unnoticed. Apparently, the probability of detecting these types of changes decreases when the spectator is actively involved in the trick (Smith, 2015). A viral video of the magician and psychologist Richard Wiseman, published in 2012, the “Color Changing Card Trick”, includes many examples of changes that go unnoticed by those who see it for the first time. As the author himself says at the end of the video, the presentation is not actually a card trick, but a very powerful demonstration of change blindness (https://youtu.be/v3iPrBrGSJM). John Henderson and Tim Smith, researchers at the University of Edinburgh, analyzed with an eye tracker the spectators’ gaze and fixations while they were watching Wiseman’s video (https://youtu.be/8wxbeEuGW00). Those who saw the video for the first time and did not notice the changes moved their eyes and explored the same parts of the scene as those who had already seen it and, therefore, already detected them. This confirms that what prevents naive observers from detecting the changes is not concealment or physical distraction, but the limits of attention and short-term memory that normally operate when we observe any natural scene. Although in a different study, Barnhart & Goldinger (2014) have observed the opposite result, that eye-movements during the critical event of a numismagic trick showed different patterns for participants, depending upon whether they saw it or not.

A third phenomenon related to the illusion of continuity gave rise to the classic magic concept of sleight-of-hand. Centuries ago the magicians discovered empirically that “the hand is faster than the eye”, that is, manipulating speed could make their maneuvers invisible. This is how, sometimes, cards, coins, balls and other gadgets are hidden or concealed in front of the eyes of the audience without them noticing. For example, the numismagic of the late twentieth century was performed with very fast maneuvers, such as the catapult of coins from one hand to the other; only after the advent of new types of gaffed coins, slow motion numismagic became possible (Gea, 2018). The speed of movements in magic was studied scientifically for the first time in 1893, thanks to the support of the newly founded French Association of Prestidigitators presided by the magician and director of theater and cinema Georges Méliès. Méliès facilitated that two reputed magicians of the time, Arnould, a great professional mnemonic, and Raynaly, a stage magician, collaborate in the experiments of the psychologist Alfred Binet. Binet studied the magicians’ maneuvers with a novel development (chronophotography) that allowed him to take about 10–15 photographs per second, and concluded that the effectiveness of many magic effects laid not only in the precision of the movements, but also in their speed, due to the inability of sight to perceive such rapid changes (Binet, 1894; Thomas, Didierjean & Nicolas, 2016).

#### Fluency and contrast

“If the audience feels impelled to analyze why you did something, you’ve already lost the battle” (Ortiz, 2006). What we have already experienced in the past is easier to process in the present. It is said that it has more fluency (Alter & Oppenheimer, 2009). And on the contrary it is also true, things that are easier to process, those that are more fluent, generate an illusory sense of familiarity, even if we have never experienced them before (Whittlesea, 1993). Processing fluency biases our perceptions and judgments. We are more likely to judge easy-to-read statements as true (Reber & Schwarz, 1999). Even jokes written in a more readable font seem funnier (Topolinski, 2014). And art works that are easier to understand seem more esthetically pleasing (Reber, Schwarz & Winkielman, 2004; Leder & Nadal, 2014). In sum, high perceptual fluency increases the experience of positive affect; and, as a consequence, greatly influences how we interact with others and with our surroundings (Reber, Winkielman & Schwarz, 1998).

The flip side of processing fluency is contrast. Brain circuits are very sensitive to contrast, that is, the differences that emerge when comparing similar objects. A classic example in vision is the degree of difference between the lightest and darkest parts of a picture that drive On-and Off-center cells in the early visual pathway (Hirsch et al., 2015). This capacity to preferentially process relative differences has been extensively used in art to capture attention and engage the audience; for instance, when juxtaposing dissimilar properties, such as color or tone in a painting, or when introducing sudden emotional shifts or unexpected turns in the plot of a movie, a novel or a play. And it also governs the visibility or invisibility of objects in magic effects. Gibson (1982) suggested that the allegedly impossible magic appearances and disappearances of objects work only because the magician hides the visual transitions that are generated in the process.

Fluency and contrast also modulate the attentional state of the audience to hide or highlight affordances. Thus, we discuss fluency and contrast only in terms of the external life of the magic effect and how they can be manipulated to influence the conscious experience of the spectator. Under this heading, we group all those resources that the magicians deploy to generate or eliminate salient features and moments associated with their maneuvers so that they do not raise suspicion and do not interfere with the intended progress of the effect. When processing fluency is high and contrast at its minimum, we pay little attention and analyze situations only superficially, as long as our interactions with the environment continue to progress smoothly. On the contrary, when processing fluency is low, contrasting situations abound and our interactions with the environment require more effort. We become more aware of the circumstances, pay more attention and tend to be more analytical to solve any problem that may arise (Alter et al., 2007; Song & Schwarz, 2008; Winkielman et al., 2003, although see Meyer et al., 2015). As we will expose in the next cognitive phenomenon, during a magic effect, magicians must manage the attention of the audience at all times to be able to guide them inadvertently and without suspicion towards the impossible outcome. Occasionally, this might require to intentionally create a significant contrast between different elements of the game, be them physical or argumentative, to capture attention during key instants of the expositive phase. But more often than not, magicians need to work in the opposite direction, actively preventing the secret maneuvers of the inner life from attracting attention, hiding or manipulating affordances and effectively increasing processing fluency and avoiding contrast with the external life so that the games progress smoothly until their shocking ending.

This absence or avoidance of contrast is crucial to strengthen a presentation of the magic effect that the public considers logical and predictable. Neglecting these aspects causes low quality effects with a high risk of ruining the magic outcome. This is so because, under these conditions, processing fluency is very low, the audience loses the thread of the presentation and begins to ask themselves questions. In a magic effect, if what the public observes happens according to what is predictable, processing fluency is high, attention is relaxed and everything supposedly superfluous is discarded. Both the design and the presentation of a magic effect in front of an audience have to be aligned with the affordances that the magician wants the spectators to perceive; any mismatch would attract attention. To paraphrase Gibson, a magic affordance “looks” in both directions, at the magician’s maneuvers and at the audience, is proper of that interrelation and does not exist outside of it (Gibson, 1979).

Magic theorists such as Darwin Ortiz or Arturo de Ascanio have written reference treaties exposing the rules and conditions to optimize this necessary avoidance of contrast in the presentation of magic effects (Ortiz, 1999, Etcheverry, 2000). In the presentation of an effect, theorists emphasize the importance of clarity both in the structure of the game and in the argument or plot of the presentation. At the same time, they highlight the importance of naturalness in everything that the magician explains and does. For this reason, for example, in magic with ropes the magicians strive to make the knots as anyone would, the magician’s gestures, maneuvers and actions during the effect must be completely natural and fully justified. This must be so even when magicians intend to manipulate non-existent objects (Cavina-Pratesi et al., 2011). In this sense, experience plays a major role in the quality of deception, as has been experimentally demonstrated (Phillips, Natter & Egan, 2015). Along with clarity and naturalness, the importance of coherence and justification of all actions and movements involved is also stressed, since both confusion and unjustified movements raise suspicions and make the presentation thread lose. They also refer to the “economy” of the actions, in the sense of avoiding steps or movements that are superfluous. Finally, theorists emphasize the timing of the maneuvers and procedures—adequate synchronization to do everything at the right time—as well as the rhythm (cadence of the acts), all with the goal in mind of maintaining control over the audience’s attention. Every action is in service of a common goal: not creating contrast and increasing processing fluency to not raise suspicions, and leading the audience smoothly towards the impossible outcome without asking questions. In sum, creating a plausible stream of affordances in the “external life”.

Magicians have devised concrete techniques to avoid contrast and contribute to the logic and predictability of the presentation of the magic effect. These techniques include the use of ruses and feints, illusory correlations and the concept of familiarization or “conditioned naturalness”. The use of feints or illusory correlations is very frequent in magic with coins. For example, some techniques of false deposit of coins begin with false maneuvers, when the magician passes a coin from one hand to another, first they do the action without cheating, which is the pre-conditioning phase, and then, when repeating the action, the trick, the false deposit, which leads to the “disappearance” of the coin is inadvertently added. As for illusory correlations, for example, when the magician needs to reinforce that in one hand or in another place there is more than one coin (especially when this is not true) the magician manages to produce the noise of the supposed coins entering into contact (as in the classic effect named “Click pass with coins”). “Familiarization”, a concept already described by Dessoir at the end of the 19th century or “conditioned naturalness”, as Ascanio later coined, refers to a special form of fluency, a kind of conditioning in a short space of time, where it is sought to normalize, always by priming and repetition, something that in any other context would contrast and attract attention (Dessoir, 1893; Etcheverry, 2000). For example, when magicians need to grab the deck of cards in a rare or unusual way at a given time, they prefer to condition the public already from the beginning of the effect with this uncommon grip.

Finally, it must be considered that contrast is not detached from context, therefore the circumstances through which the minimization of contrast is structured in magic always depend on the social and circumstantial context of the presentations. The setting, the atmosphere and the type of public are decisive. It is not the same to do magic in the street than in a closed place, in a noisy or silent environment. In short, the same outcome can be very magic or completely anodyne depending on the scenario. Context is thus constitutive not just enabling (Gomez-Marin & Ghazanfar, 2019).

#### Attention

Magicians have adopted the term “misdirection” to refer to one of their best tools: the control of attention. Through misdirection they can regulate which affordances are consciously perceived and which ones are missed during a magic routine. The term was consolidated in magic reference texts at the beginnings of the 20th century, like those of the legendary Nevil Maskelyne & Devant (1911) and Tarbell (1927). In fact, magicians are true specialists in the subject. They have developed diverse techniques to control the spatial and temporal aspects of attention, including not only where we focus our attentional resources in space and time, but also how to deviate and divide them (Wonder, 1994).

Let’s start with the spatial capture of attention that is, usually followed by its overt deviation. Magicians use their gestures and gaze to direct the public’s attention to a particular place or focus away from the method. The aim of the deviation in magic is to create new areas of attention in order to perform some maneuver outside these areas of interest. In this sense there are several procedures for attentional exogenous capture (passive or bottom-up) and subsequent overt deviation (known among magicians as “physical misdirection”), such as the introduction of contrasting stimuli, the so-called by Ascanio “priority movements” (Etcheverry, 2000), and the use of social cues.

The most used contrasting stimuli for exogenous attention capture are sounds (such as rhythms, changes in tempo or other musical scores), and surprising appearances, such as the production of some striking object in contrast to the main storyline. Beyond the trick itself, this is also the result, for example, of the classic rabbit that comes out of a hat, the visual impact of the igneous flash paper, or the sudden change in color of a handkerchief or the back of a deck of cards.

Magicians have also learned empirically that not all movements have the same attentional valence. A large movement can cover a small change (Suchow & Alvarez, 2011), and this, in the magic jargon, has been translated as “priority movement”, a concept that includes either the first movement that is, performed or the movement that has greater amplitude. The study of movements in magic effects has received significant recent attention. In particular, it has been experimentally tested whether curved movements capture more attention than rectilinear ones (Otero-Millan et al., 2011; Tachibana & Gyoba, 2015), if some locations and directions of movement in space are more salient (Stone, 2011), or the influence of relative speed in simultaneous trajectories (Hergovich, Gröbl & Carbon, 2011).

Both for the recruitment and for the control of attention, social cues are a strategic resource often used in magic effects. Among the different aspects of nonverbal communication, the magician’s gaze plays a central role to such an extent that, in many magic effects, the control of attention depends almost exclusively on gaze control, always with the reinforcement of an appropriate body language. One of the most celebrated “misdirection” techniques is the “crossing of gazes” introduced by the magician Tony Slydini. This technique is based on using interacting gaze movements to deviate attention from the existence and handling of a small object in one hand (Tamariz, 2005). Experimentally, it has been reported that social cues, beyond gaze location, can manipulate the audience’s attention effectively (Kuhn & Tatler, 2005; Kuhn et al., 2008a; Kuhn, Amlani & Rensink, 2008b). In general, social cues strengthen the effectiveness of the magic effects, can be imposed on explicit instructions and allow manipulating the audience’s expectations (Cui et al., 2011; Hergovich & Oberfichtner, 2016; Kuhn & Land, 2006; Kuhn, Tatler & Cole, 2009; Kuhn & Teszka, 2015; Kuhn et al., 2016; Kuhn & Rensink, 2016; Rieiro, Martinez-Conde & Macknik, 2013; Scott, Batten & Kuhn, 2019; Tachibana & Kawabata, 2014; Thomas & Didierjean, 2016a, 2016b; Tompkins, Woods & Aimola Davies, 2016).

In contrast to exogenous capture, endogenous capture of attention (active or top-down) is linked to covert deviation (or “psychic misdirection” in magic). The covert deviation is achieved when the focus of attention of the audience shifts away to another place or thought, without necessarily having to mediate a change in gaze (“you look, but you do not see”). This covert deviation of attention is very important in some close-up magic effects and, in most cases, it is achieved through the use of dividing techniques. Divided attention, and its adverse consequences, such as inattentional blindness (Simons & Chabris, 1999), is an increasingly current phenomenon, since human beings have adopted the use of mobile phones and other portable and wearable devices. Accidents associated with distractions related to the use of phones, texting or consulting the GPS while driving a car, or even while crossing busy streets, are now issues of social concern. Magicians have acquired a secular experience with the cognitive limitations derived from dividing attention. Magicians use the division of attention both to hide methods and to hinder the reconstruction of the effect by the audience. With divided attention everything is a little easier for the magician, the spectators cannot assimilate everything that is, happening on the scene, nor do they find out about certain maneuvers necessary for the method. In magic, the most used techniques to divide attention are the introduction of sudden distractions and demanding tasks.

Sudden distractions are achieved, for example, by asking extemporaneous questions. The magician Ascanio coined the concept of “obnubilant question”, to describe these techniques of attentional interruption that are used to perform necessary maneuvers that need to go unnoticed, and that under normal conditions would have been very evident (Etcheverry, 2000). Alfred Binet described them with the following example: “Suddenly I ask the spectator sitting in front of me: Do you know how to count to sixty? The spectator looks at me, self-conscious, not knowing how to answer the question; the others look at him with a smile; it only lasts a second, which is enough to peek at the card” (Binet, 1894). The introduction of demanding tasks is also a common feature of magic shows, especially used with those spectators invited to participate in effects. For example, if a spectator is asked to both find a chosen card in a shuffled deck, and simultaneously find out its position by counting the cards face up one by one, the situation is demanding enough that most of the spectators would not realize that the cards in the deck are sorted in a particular way or even repeated.

Endogenous and exogenous capture of attention do not function as isolated compartments, rather they usually interact with each other during natural vision. Smith, Lamont & Henderson (2013), found that during a cardmagic trick endogenous factors strongly control attention during dynamic viewing and can override exogenous influences even to the point of misrepresenting the visual scene.

We have mentioned that the temporal control or continuous direction of attention is as important as its spatial control. The attention of the audience fluctuates throughout a show. Spectators spontaneously look for moments of relief and can be easily distracted. The goal of the magician is to temporarily capture attention, control it during the entire expository phase of the effect, and ensure that during this period the spectators’ trains of thought do not proceed on their own. Temporal control of attention is a goal in itself during a magic effect, not only to prevent the audience from discovering the method, but also to ensure that they understand the entire expository phase so that no undesired contrast occurs and processing fluency remains high. Beyond naturalness and clarity, timing and rhythm of the maneuvers are key for the continuous direction of attention since they create different attentional hotspots and areas of interest in the service of the magical effect (Barnhart et al., 2018). If the temporal control of attention fails, the audience is distracted, does not follow the magician, and the magic climax is ruined.

For the continuous direction of attention, magicians use all kind of personal, plot and stage resources. Among personal resources, everything related to the character that is, introduced, its appearance, outfits and its way of speaking and presenting are relevant. Plot resources, beyond the quality of the narrative that we have already highlighted, can be very varied. A common example is the introduction of expectations, even those of fake failure, which help to continuously increase curiosity as the effect progresses. And, as for the scenic resources, it is necessary to emphasize the importance of lighting and music in certain effects. Specially in the field of magic of “Grand illusions” (stage magic with large objects) music is fundamental to capture, control and synchronize the attention of the audience and to punctuate or highlight very specific moments along the way that enhance intermediate outcomes or the power of the final climax.

The continuous control of attention is a key requirement and probably a unique and characteristic feature of magic. The illusion of impossibility is never achieved if the spectator sees the effect halfway; one has to follow the presentation of the effect completely, from the beginning to the end. Magic, and perhaps cognition in general, are serial, not sequential, phenomena and this contrasts sharply with the way we design cognitive experiments in the lab based on a sequential accumulation of trials. Therefore, magic is a very demanding task both for the magicians and spectators, and that is, why magicians have become true experts in the collective control of attention, at once, in real time, and for everyone. However, since temporal control of attention is a function directly dependent on the very limited short-term memory, this continuous demand has other derivatives and the formation of memories of the magic show is subject to great stress. The demand for continuous attention, coupled with the excessive information that is, generally provided during the exposure of various effects, drains and saturates the public, and may even affect the ability to correctly perceive a scene (Ling & Carrasco, 2006). The father of modern magic, the legendary magician Robert-Houdin, already warned about this, prescribing that magic shows should have a limited duration (Robert-Houdin, 1868).

Finally, moments of deactivation of attention (known as “off-beat” moments, a term that was borrowed from music) are also very important for the purposes of magic. Magicians achieve a deactivation of attention, for example, when they induce collective laughter through humorous gags or collective clapping, including post-climax applause, that occurs during some games containing various effects. The complete deactivation allows the magician to make some arrangement or manipulation (change of deck, loads or downloads, etc.), often in plain view without the audience being aware of it.

#### Perception

Magic effects interfere with perceptual phenomena, understood here as the cognitive processes of close-loop inference and interpretation that emerge to compensate for the limitations of capacity and slowness of cerebral processing in highly dynamic environments (Ahissar & Assa, 2016; Friston, 2018). Perception operates generating adaptive predictions and affordances based on context and past experiences from memories (Gibson, 1979; Clark, 2013). The fact that the human brain anticipates the future is something that the world of magic has learned empirically and that considers in the production of magic effects.

Indeed, most magic effects rely on breaking expectations and have a totally unexpected outcome. When the audience is not in a position to anticipate what will immediately happen, the capacity of the magician to have a continuous control of attention is greatly increased. The brain is very good at detecting novelty, when something is observed for the first time it captures attention very efficiently. Thus, magicians never reveal in advance the full nature of an effect, so that the spectator does not know where to focus attention. On the contrary, when the audience can predict what is going to happen next, they stop making the same effort, being able to turn attention inconveniently towards other details potentially ruining the effectiveness of the effect. This reduction of the attentional effort is consistent with the perceptual fluency heuristic mentioned above (Whittlesea & Leboe, 2000).

There is a related precept in magic according to which it is necessary to avoid doing effects with the same method in the same presentation, or the same method more than once in the same effect. Indeed, there is a tendency to consider that things that are repeated over and over again have the same cause. A continuous repetition of methods encourages the audience to test any perceptual hypothesis that has been raised before and be convinced that what is repeated is always done in the same way. If the audience sees exactly the same over and over, it will be progressively better able to anticipate what will happen next and it will be able to grasp inconvenient details. The magician will lose the ability to control the attention of the public and the effectiveness of the magic outcome will be jeopardized. For this reason, in effects in which actions are repeated, magicians tend to continually change methods. Consistent with this, Kuhn and colleagues have experimentally confirmed that manipulation capacity is extinguished by repetition, and that, in general, prior information about the magic effect being performed significantly increases the probability that the participants detect the method (Kuhn & Tatler, 2005, Kuhn et al., 2008a; Kuhn, Tatler & Cole, 2009; Kuhn & Findlay, 2010). Although some simple maneuvers, such as those based on the false deposit, seem to be more resilient to repetition (Cui et al., 2011; Otero-Millan et al., 2011).

The inferential, automatic and unconscious nature of perceptual processes leads to consistent predictions that magicians can hijack to construct surprising effects. For example, when partially hidden objects are presented to an audience, the visual system automatically and immediately fills in the invisible parts of the objects, even when the information is very scarce, a phenomenon known as amodal completion (Kanizsa, 1985). This phenomenon follows gestalt laws, it is difficult to master for a magician but it provides great advantage in certain effects, especially in magic using ropes (Barnhart, 2010), in that of bent spoons or in the Chinese rings. Also, in cardmagic, there is amodal completion in some moments of the effects of the “torn and restored card” (i.e., Guy Hollingworth version by David Regal’s “Piece by Piece”), or in those of “linking cards” (i.e., “The Immaculate Connection” by Paul Harris). In all these cases, the magician’s hand acts as a screen, partially covering the objects at certain times, in circumstances in which the public tends to interpret what is hidden in a very different way from reality. In Chinese rings, when the magician shows the ring while covering the gap that breaks its continuity, a complete ring is automatically perceived. What is behind the screen is never questioned, escapes conscious control, because the fill-in process acts by default, never fails and it is immediate. The same effect can be repeated again and again. A very striking case is that of manipulating balls, where the illusory experience persists even when the spectator knows that semispherical shells are used instead. The three-dimensional curvature of the visible surface of the object is sufficient for it to be perceived as a sphere, the audience remains with this perceptual solution and no other alternative is explored (Ekroll, Sayim & Wagemans, 2013; Ekroll & Wagemans, 2016; Ekroll et al., 2016).

In general, partial concealments in magic allow spectators to interpret incomplete information and automatically perform perceptual filling-in. Sometimes, this perceptual completion is based on assumptions constructed through experience in relation to environmental regularities. Following Barnhart, one of many assumptions used by magic spectators is symmetry, both in static situations and in sequences of actions (Barnhart, 2017). In addition, amodal perception resists repetition very well, unlike the capture and active deviation of attention, whose effectiveness decays very rapidly in successive passes (Ekroll et al., 2018). In magic, concealments based on amodal perception have the advantage that they invoke automatic assumptions of the visual system that are not suspicious, that do not induce the spectator to “rewind” or think about the method behind the effect, they do not contrast with the intended flow of the game. Ekroll and colleagues recently claimed that these automatic inferences are “cognitively impenetrable perceptual mechanisms”, that do not reach the conscious level; and they go on to suggest they must play a central role in many magic effects that has been often ignored because of the disproportionate weight traditionally assigned to “misdirection” and other forms of attentional control (Van de Cruys, Wagemans & Ekroll, 2015; Ekroll & Wagemans, 2016; Ekroll, Sayim & Wagemans, 2017).

The flip side of amodal completion is amodal absence, the phenomenon by which what we do not see does not actually exist (Ekroll, Sayim & Wagemans, 2017). This is the basis of many concealments, such as hiding coins in one hand, or the convincing illusion of empty space surrounding levitating objects and persons, and vise versa (Øhrn et al., 2019). Like amodal completion, amodal absence is also considered a perceptual, automatic and unconscious illusion (Andersen et al., 2017; Ekroll, Sayim & Wagemans, 2017).

#### Episodic memories

An integrated, very interesting perspective about the role of memories in magic has been originally proposed by Quian Quiroga (2016). Magic effects interact with the memory processes of the audience at all levels, from iconic and working memory, which we have already discussed in previous sections, to episodic memories. The construction of magic effects includes techniques for the manipulation of episodic memories, either at the service of the effect itself or to prevent the public from reconstructing the method afterwards. Already in 1973, the renowned Spanish magician Juan Tamariz wrote: “The magician has to know how to cause gaps in the memory of the spectators to make them forget what we want for the magic effect, or make them believe they remember things that did not really exist…” (Tamariz, 1988). Accordingly, some magic techniques have been developed to distract, affecting the codification and consolidation of memories, misinform, hinder recall and promote forgetfulness.

For the promotion of forgetfulness, common techniques are related to the deviation and division of attention, and the so-called “time misdirection”. When a magician diverts attention during an effect, the rationale is that there is a weakening of the codification and consolidation of the information captured at that moment. One can also promote forgetting by dividing attention through highly distracting comic gags, or tone outings with extemporaneous questions. On the other hand, “time misdirection”, the equivalent to Ascanio’s “parenthesis of forgetfulness”, introduces a temporal (and sometimes spatial) distance or delay between the moment of the method and that of the effect resulting for that method (Etcheverry, 2000; Fraps, 2014). There is already some experimental evidence on the relationship between the time separating method and outcome and the potency of the magic effect. This relationship follows an inverted U shape, that is, the effective times are intermediate, neither too short nor too long (Beth & Ekroll, 2015).

Misinformation can affect recapitulation by distorting long-term memory recall. Magic techniques take advantage of the fact that the entrance door to episodic memories, short-term memory, saturates easily. These techniques generally create overwhelming situations in which the audience is flooded with an excess of information. The magician Juan Tamariz is known for having developed a special ability to induce false memories in his audience, especially when recapitulating the game, as he makes a point of explicitly detailing having shuffled and cut the cards in a way that, in reality, was never done. The magician Dani DaOrtiz performs an effect, called “The One”, in which, after discreetly forcing a card to a spectator, he manages to get the audience to think that the card has been “thought” freely by the spectator instead (DaOrtiz, 2011). It has not yet been investigated to what extent the memory errors induced by Tamariz or DaOrtiz are homologous to the false memories described in the scientific literature (Loftus, 2003); what is indisputable is that their magical routines based on misinformation generate similar effects in a much shorter time span, the few seconds or minutes that a game lasts, and without the need for repetitions or reinforcements.

When spectators grasp how magic works then they have a feeling similar to an “aha!” moment. Beyond the distinct frequency of “aha!” moments, it seems that these are correlated with the subsequent memorability of the magic effect (Danek et al., 2013, 2014a, 2014b; Hedne, Norman & Metcalfe, 2016). It has been proposed that audiences are “biologically” impelled to discover the secret behind the method of a magic effect, seeking to regain the cognitive control that has been disrupted by the magician (Prevos, 2013). Throughout this inevitable search for solutions, magicians are aware that the public has “aha!” moments when they believe they have deduced the method. Whether they are right or wrong does not really matter, these moments can either way ruin the magic effect at more than one level, even if they lead to the wrong deductions (Ortiz, 1999). Although some spectators may realize the moment when the magician executes the method, this rarely helps them solve how the magic effect occurs (Demacheva et al., 2012). Still, when an idea-solution appears in mind as reasonable, it is very difficult to consider other alternatives, a phenomenon known as the Einstellung effect (Bilalić, McLeod & Gobet, 2010). Therefore, anything else that at this point the magicians present to us will be likely filtered out, affecting the experience of the game. In addition, most naive spectators tend to overestimate the ability of other members of the audience to deduce the method behind the effect, especially if they themselves believe they have discovered it (Ortega et al., 2018). All this is relevant for a magician and in order to manage the “aha!” moments during their shows, magicians know that the first requirement is to avoid contrast, to increase perceptual fluency, making sure that nothing attracts abnormal attention throughout the presentation of the effect. To control the emergence of “aha!” moments and the audience’s intuitions, magician Juan Tamariz has proposed techniques to introduce “false clues” at relevant moments designed to create false expectations or to subtly suggest controlled wrong solutions (Tamariz, 2011). So far, few studies have begun to experimentally validate these proposals (Thomas & Didierjean, 2016c; Thomas, Didierjean & Kuhn, 2018).

Tamariz (2016) gives great importance to what the magician can do during the show to control the memories that the audience take home, both to distort and specially to magnify the magic experience. We wonder if these magic memories are similar to those acquired in especially emotional circumstances (flashbulb memories), in which the vivid memory of the experience does not guarantee the trustworthiness of its details (Hirst et al., 2015). Along these lines, memorability studies on supposedly paranormal experiences (some published more than 130 years ago) show that memories are very unreliable, and that, depending on the circumstances, there is a propensity to remember events that have not happened (Hodgson & Davy, 1887; Besterman, 1932; Wiseman & Morris, 1995; Wilson & French, 2014). Subjects are also susceptible to manipulation through suggestion and instructions (Wiseman, Greening & Smith, 2003; Wiseman & Greening, 2005; Wilson & French, 2014). We believe that these studies can be a good reference to plan the necessary and yet non-existent studies on the subsequent memorability of magic shows. We have recently made a first attempt in that direction and showed that the memory of a magic trick decays over time as does that of other episodic memories. However, the serial-position differences in memorability, recency effects, that were evident after the show were no longer present later on, suggesting that short-term memory gains do not translate into the long-term (Bestue et al., 2020). This preliminary work is, to our knowledge, the first scientific study of the memorability of magic tricks; and it illustrates the power that magic holds to study memory, and cognition in general, in real-world environments.

#### Subliminal effects during magic tricks

The inner life of a magic effect, even when it is not consciously perceived or processed by the audience, might leave an unconscious, weak, and ephemeral trace. Shalom et al. (2013), have described physiological changes, transient pupil dilations, related to the subliminal perception of a card that is, in view slightly longer than the others, during a rifle in front of the spectator. This maneuver, which goes unnoticed because it falls below the subjective threshold for conscious perception, nevertheless influences the spectator’s decisions thereby helping the magician in forcing the intended card. It is also well known that, through priming—a phenomenon related to implicit memories—there is the possibility of influencing attitudes, perceptions and choices unconsciously (Dehaene et al., 1998). Magicians are masters conditioning responses and surprising the public by “predicting” the choices that a spectator will make. There are experienced magicians even capable of achieving priming effects during the seconds or few minutes that the presentation of an effect would last, influencing the subsequent choice of the audience through direct or indirect cues (for example, color, suit, card value, etc., see next section). It is difficult to think of a more ideal scenario to investigate subliminal perception, and how subtle interpersonal differences can influence memory processes and conscious reasoning in ecological conditions.

Subliminal perceptions during a magic trick, however, have their own dark side. In words of the Spanish magician Miguel Ángel Gea, “sometimes the audience feels the trick even if they do not perceive it” and this might end up having an impact on the experience of the magic effect, or even its potential reconstruction afterwards (Gea, 2018, see also Kawakami & Miura, 2017). This could explain why the same magic routine, performed by different magicians, does not necessarily achieve the same magic potential, even when it is always allegedly done in the same way, using the same techniques, and with the same procedures. The line between success and failure is, in this case, a very thin one.

#### Intuitive decisions (forces, manipulating decisions)

When magicians interact directly with the audience, especially when inviting spectators to participate in any of their effects, they often ask them to answer questions or make decisions. Magicians look for these responses to be as fast and automatic as possible (intuitive decisions, according to Gigerenzer (2007)). They don’t want the opposite, reflexive answers that can lead the audience to process relevant information for the subsequent reconstruction of the trick. They just want to control the audience’s attention and influence their decisions. Magicians have developed very robust techniques to induce choice and manipulate responses. In the magic slang they are called “forces” and they seek to overwhelm the spectator by not giving them time to think or reflect, controlling all their reactions. The main characteristic of a good “force” is that the spectator always considers that they have made a free choice (Shalom et al., 2013; Olson et al., 2015; Olson et al., 2016). Even in those cases, when they subsequently rationalize the causes of their decisions, they end up justifying them using fallacious arguments, which the magician knows have not been part of the choice set, a situation that has been dubbed "choice blindness" (Johansson et al., 2005, 2006).

There is a wide spectrum of forces, from automatic techniques (which always lead to the desired result), to techniques that seek to obtain the most probable answers (the result of which is not sure a priori, and which are known in magic as “psychological forces”). In cardmagic, many automatic techniques are based on mathematical methods. There are also more complex techniques, which depend on the skill of the magician, such as the so-called “magician’s choice”, in which the artist controls and varies the type of questions he asks the spectator as he chooses between different options, always arriving to the solution originally designed by the magician. Some forces are also based on the use of visual prominence; such as, for example, exposing a given card for a longer time in a subtle way, a subliminal influence that is, also rarely noticed by participants (Shalom et al., 2013; Olson et al., 2015). In the field of riskier forces, the reference in cardmagic is the so-called “classical force”, a technique that has been practiced identically since at least the nineteenth century (Triplett, 1900) and whose effectiveness, which can be of 100%, is totally dependent on the training and experience of the magician. It consists of inviting the spectator to choose “freely” a card from the deck offered by the magician after presenting all the cards in a fan. The selection appears to be free, although in reality it is a forced delivery. Essentially, the key to this force lies in the magician’s control of the spectator’s reaction time. One prominent variant that requires the use of social cues is DaOrtiz’s “force at the stop”, in which spectators are invited to choose a set of cards as he sequentially leaves them face down on the table (DaOrtiz, 2010, 2011).

There are other techniques of “psychological forces” where situations or questions arise in which the magician expects the spectator would have a very specific type of reaction or response. Since it is not possible to obtain the expected response with 100% certainty, in these cases the magician always has a “way out” or plan B to solve the situation. In these forces, sometimes a previous “priming” of the response is introduced (see “Subliminal Effects During Magic Tricks”). In others, the magician risks the spectator giving the most probable automatic response. If several cards or piles of cards are set up on a table, magicians know that right-handed people tend to choose the card or pile placed in second position counting from their right. Other authors have already described a positional bias influencing choice using everyday objects, but in this case, the selected position is the first, not the second, from the right (Nisbett & Wilson, 1977). Kuhn, Pailhès & Yuxuan (2020), suggest that this preference for the second position is caused by a similar positional bias towards the card that it is easiest to reach from the point of view of the spectator. However, in magic shows, sometimes the magician deals the cards on the table at a slant, if the positional bias was the only responsible for people’s choices, the selected card in this case would be the one closest to the spectator, that is, the first one from the right. However, also in this case, the most commonly picked card is still the second one from the right. An alternative explanation is that we do tend to pick the most accessible card (which is the first card in the slanted version, or the rightmost card in the parallel row), but since we intuitively know that the magician will try to deceive us, we often decide to not go for the easiest card and select the next one instead. This explanation would also be more consistent with Nisbett and Wilson’s results.

In other instances, magicians seek to obtain specific verbal responses, always under pressure and creating very specific contexts. For example, frequent or prototypical word responses stored in semantic memory, such as “a canary is… yellow”. In this line, many magic effects have been designed based on asking the audience to name colors, numbers, geometric shapes, objects; it has even been described that some expected answers are different according to the genre or the context in which they are formulated, although none of it has been experimentally tested (Wright & Larsen, 1936). Magicians usually go after prototypical or representative responses of a certain category or class. Among animals, a cat and a dog are more frequent or representative responses than a kangaroo, but magicians (like linguists) also know that the words characteristic of a given category vary widely in different cultural contexts. If in Spain we are asked about a vegetable, we may say lettuce or tomato, but this might not be the first vegetable that comes to the mind of the north-American or the Chinese, for example. This type of “psychological forces” likely depend on the heuristic of availability (Tversky & Kahneman, 1973).

When the magician asks a spectator to say a number between 5 and 10, or even between 1 and 10, the probability that the answer is number 7 is very high. This has been exploited in magic for centuries (Binet, 1894; Wright & Larsen, 1936; Kubovy & Psotka, 1976). Olson, Amlani & Rensink (2012), have experimentally studied the perceptual characteristics of the cards of the poker deck, observing that some are visually more accessible, others are better remembered and some are chosen more often than others, being among the most appreciated the Ace of Hearts, the Queen of Hearts (mainly in men) and the King of Hearts (mainly in women). There are still no studies that have reproduced these observations, although they are compatible with the experiences available in the world of cardmagic. Beyond the search for the most likely answer, magicians have also empirically learned the importance of framing. Although they may seem similar instructions, the results of a card trick vary greatly depending on whether the magician instructs the spectator to think, choose, indicate, point, touch, or take a card.

## Conclusions

The first formal research experiments to unravel how magic works date back to the end of the 19th century (Lachapelle, 2008). Among them are the pioneering studies of psychologists such as Alfred Binet or Joseph Jastrow (Binet, 1894; Jastrow, 1896). At the beginning of the 20th century, scientific interest in magic declined completely as the cinema appeared and gained wide popularity. It has not been until very recently that the scientific community has regained the interest for illusionism. The leadership is of psychologists that are also magicians, among them Gustav Kuhn and colleagues stand out. He was the author of one of the first experimental studies on the perception of a magic effect and the role of divided attention (Kuhn & Tatler, 2005) and of a recent extended review on the “Science of magic” topic (Kuhn, 2019). In Kuhn et al. (2014) published a new theoretical proposal in the form of a taxonomy of magic effects which they divided in three main classes of misdirection techniques (perception, memory and reasoning). Recently, Rensink and Kuhn have used a similar approach to present a framework for using magic effects for studying the mind (Rensink & Kuhn, 2015). An approach that was followed by other authors as well (Lamont & Wiseman, 1999; Thomas et al., 2015). An alternative paradigm, one that is, somewhat closer to the one we present here, was introduced independently by the group of Susana Martínez-Conde and Steve Macknik (Macknik et al., 2008), who proposed the need for a more causal approach to magical effects, more based on their neurobiological bases.

For neuroscience to benefit from the polished methodology, overwhelming successes, and unique perspective of magic, it is essential that the joint work between magicians and scientists continues. This is a cross-disciplinary area of research for which there is an immeasurable pending journey. Indeed, since the end of the 19th century to date, the actual experimental articles (excluding revisions and editorial works) in which magic effects have been used either as a resource or as a research goal do not reach a hundred (Tompkins, 2020). It is a ridiculous figure from all points of view. One only needs to compare it with that of any other scientific area of study. Therefore, it would not be an exaggeration to claim that, when it comes to the relationship between magic and science, everything is virtually yet to be done. We hope that the work presented here contributes to arise greater interest in magic amongst cognitive scientists writ large.

**Figure 3:**
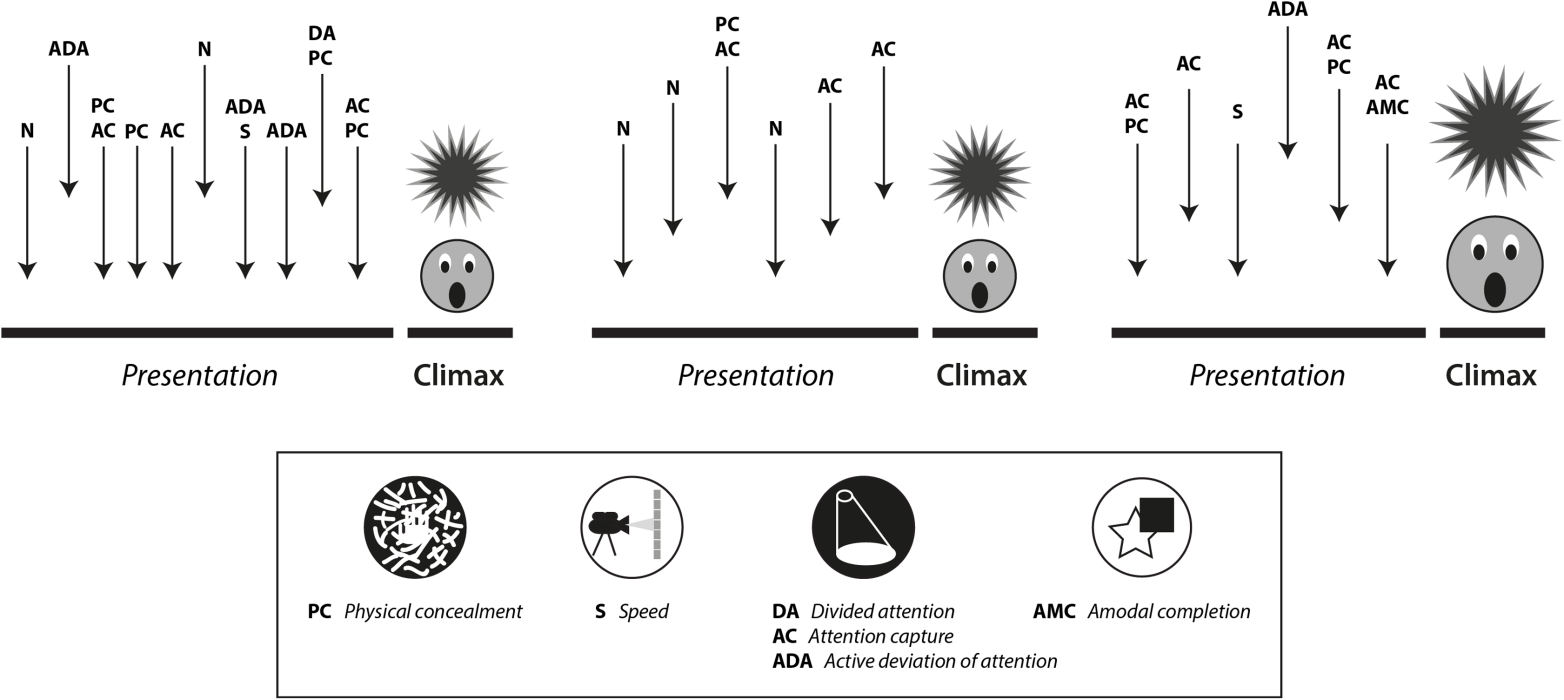
Deconstruction of a magic trick (“Homing card” effect by Francis Carlyle according to the version of Tino Call). The figure depicts that the game takes place in three phases, each with its own climax. In the internal life of each phase there are several maneuvers of attention control and concealment. Note that no phase is identical to each other (although apparently the same things happen in the first two).

Our proposal based on the concept of magic affordances and ecological cognition highlights the main pre-sensory and cognitive phenomena that magicians control and manipulate when designing and performing their magic effects (Box 1; Fig. 3). They suggest largely untrodden areas of research for which magic could contribute to a better understanding of human cognition. Let us mention a few. The lack of knowledge about the components of the cognitive dissonance that the illusion of impossibility entails stands out. Moreover, there are huge differences in the experience of magic depending on the context in which it is performed. This extends to the cultural background of the spectators. Inter-individual differences in the reactions of the magic audience can also be large. In particular, it is well known that magic is very different if it is directed at adults, children or other magicians (or even machines, Zaghi-Lara et al., 2019). Children, unlike adults, often detect many details of the methods behind the effects that have been designed for more mature audiences. This is likely due to the fact that they have not yet developed the same heuristics and do not perceive the same affordances that characterize adult information processing. For kids, every single detail is potentially relevant information. So, they also realize affordances and meanings during the presentation of the effect that adults might miss. Magicians rarely experience the illusion of impossibility because they are well versed in the art, but enjoy the technical abilities and conceptual innovations of their peers. Thus, a variety of questions emerge when considering cognition under the lens of illusionism. We propose to update and revisit the powerful arsenal that ancient magic techniques provide as a unique, and we believe untapped, tool to improve our research strategies in and specially outside the laboratory.

**Box 1:** Proposal to “deconstruct” a magic effect.

Below are the details that contain a specific magic effect, exclusively in relation to the techniques and methods based on and with cognitive consequences. Although all magic effects may contain techniques and methods from many different disciplines, in this case the pre-sensory and the different cognitive mechanisms that it includes have been identified. They are not easily standardized exercises since many magic effects that give rise to the same outcome can be constructed and materialized in very different ways throughout one’s internal life, using different materials and methods. That is, there are generally several apparently identical versions of the same effect, although they are not strictly the same because the methods used are different.
The effect chosen for its deconstruction here is the “Homing Card” by the magician Francis Carlyle. It contains several concealments and many maneuvers related to attention. As illustrated in Fig. 3, the game takes place in three phases, each phase is an effect, that is, each phase has its own outcome or climax. The main feature is that a card freely chosen by the audience and then signed travels surprisingly from the deck to the magician’s pocket. This happens in the first two phases. In the third phase, what travels to the magician’s pocket is the entire deck except for the chosen card, which is the only one that remains in the magician’s hand.
This effect is based mainly (although not exclusively) on successive captures and deviations of attention. It depends as well as on several physical concealments, including some card palms and card manipulations, that must be well executed to prevent the generation of contrast. At the end of the third phase, when the magician only holds a single card in his hand, a phenomenon of amodal completion makes the audience consider that he is actually holding the entire deck.
We present the deconstruction similar to the original, made by the magician Tino Call (see Fig. 3 and details of the effect in https://youtu.be/BVvmtv2D8MU).

## Additional Information and Declarations

### Data Availability

The following information was supplied regarding data availability: This is a literature review, there is no raw data.
